# Kinetics of specific anti-SARS-CoV-2 IgM, IgA, and IgG responses during the first 12 months after SARS-CoV-2 infection: A prospective longitudinal study

**DOI:** 10.1371/journal.pone.0288557

**Published:** 2023-07-12

**Authors:** Houda Amellal, Najlaa Assaid, Hicham Charoute, Khadija Akarid, Abderrahmane Maaroufi, Sayeh Ezzikouri, M’hammed Sarih

**Affiliations:** 1 Department of Parasitology and Vector-Borne Diseases, Institut Pasteur du Maroc, Casablanca, Morocco; 2 Aïn Chock Faculty of Sciences, Health and Environment Laboratory, Biochemistry, Biotechnology and Immunophysiopathology Research Team, Hassan II University of Casablanca, Casablanca, Morocco; 3 Institut Pasteur du Maroc, Research Unit of Epidemiology, Biostatistics and Bioinformatics, Casablanca, Morocco; 4 Viral Hepatitis Laboratory, Institut Pasteur du Maroc, Virology Unit, Casablanca, Morocco; University of Bologna / Romagna Local Health Authority, ITALY

## Abstract

Coronavirus 2019 (COVID-19) is a global health threat. The kinetics of antibodies against severe acute respiratory syndrome coronavirus 2 (SARS-CoV-2) need to be assessed, as the long-term duration of these immunoglobulins remains largely controversial. The aim of this study was to assess the longitudinal dynamics of anti-SARS-CoV-2 antibodies against the nucleocapsid (N) protein and the receptor-binding domain (RBD) of the spike protein up to one year in a cohort of 190 COVID-19 patients. Between March and September 2021, we enrolled patients from two regional hospitals in Casablanca, Morocco. Blood samples were collected and analyzed for antibody levels. We used the commercial Euroimmun ELISA for the determination of anti-N IgM, the Abbott Architect^™^ SARS-CoV-2 IgG test for the detection of anti-RBD IgG, and an in-house kit for the assay of anti-N IgG and anti-N IgA. IgM and IgA antibodies were assessed 2–5, 9–12, 17–20 and 32–37 days after symptom onset. IgG antibodies were also assessed 60, 90, 120 and 360 days after symptom onset. One-third of patients developed IgM (32%), while two-thirds developed IgA (61%). One month of symptom onset, most patients developed IgG, with 97% and 93% positivity for anti-RBD IgG and anti-N IgG, respectively. The anti-RBD IgG positivity rate remained high up to one year of follow-up. However, the anti-N IgG positivity rate decreased over time, with only 41% of patients testing positive after one year’s follow-up. IgG levels were significantly higher in older people (over 50 years) than in other study participants. We also found that patients who had received two doses of ChAdOx1 nCoV-19 vaccine prior to infection had a lower IgM response than unvaccinated patients. This difference was statistically significant two weeks after the onset of symptoms. We present the first study in Africa to measure the kinetics of antibody response (IgA, IgM and IgG) to SARS-CoV-2 over one year. Most participants remained seropositive for anti-RBD IgG after one year but showed a significant decline in antibody titers.

## Introduction

The rapid global spread of severe acute respiratory syndrome coronavirus 2 (SARS-CoV-2) began with its emergence in Wuhan, China, in December 2019, resulting in a pandemic [1]. COVID-19 is the disease caused by SARS-CoV-2 and can manifest itself with a wide range of clinical symptoms, from asymptomatic, mild infections to severe pneumonia requiring respiratory ventilation support [2,3]. Humoral immune responses play an essential role in adaptive immunity against viral infections [4]. Nucleocapsid (N) protein and spike (S) protein are two structural proteins commonly used as target antigens for serological assays.

The N protein is responsible for maintaining the viral RNA genome. The S protein, on the other hand, is located on the surface of the virus and plays a crucial role in its entry into human cells. Protein S enters the virus by interacting directly with the human angiotensin-converting enzyme 2 (ACE2) receptor via its receptor-binding domain (RBD) [5,6]. Antibodies generated in response to SARS-CoV-2 infection have the ability to target various proteins encoded by the virus, which include both structural and non-structural antigens. The highly immunogenic trimeric glycoprotein S serves as a viral target for neutralizing antibodies. IgA, IgM and IgG levels targeting the SARS-CoV-2 proteins S and N evolve rapidly within 1–2 weeks of symptom onset in COVID-19 patients [7–10]. Anti-N IgG antibodies generally appear two days earlier than anti-S IgG antibodies [11]. Several studies have shown that most patients with COVID-19 produce detectable SARS-CoV-2-specific antibodies that target the N protein and the RBD of protein S during the acute phase and early convalescence [12–14]. Almost all individuals with symptomatic COVID-19 produce anti-SARS-CoV-2 antibodies, and levels of these antibodies are positively associated with symptom severity [10,15–18]. Studies have indicated that peak antibody levels are lower in individuals with asymptomatic or mild infections [19]. It has been described that patients who died of COVID-19 showed a strong but delayed production of anti-S and anti-RBD IgG and neutralizing antibody (nAb) levels compared to survivors. This delay in antibody production was found to be associated with impaired viral control [20]. The persistence of SARS-CoV-2 antibodies has been debated, with some studies reporting a rapid decline in antibody levels as early as 3 months after infection [21–23], while others suggest that antibody responses may persist for up to 28 months after infection [24–27]. According to a recent comprehensive study examining T and B cell responses in individuals who have recovered from COVID-19, significant immune memory is generated after the infection, with approximately 95% of participants maintaining immune memory around 6 months after their initial infection [28]. Additionally, the study found that the presence of IgG antibodies to SARS-CoV-2 S and nucleocapsid (N) was associated with a lower risk of reinfection with SARS-CoV-2 up to 7 months after the initial infection [29–31]. Robust data on the persistence and long-term efficacy of the immune response are essential for a full understanding of pandemic progression and post-pandemic scenarios, particularly with the emergence of new variants [32,33]. Characterizing the magnitude and persistence of humoral responses over time in people symptomatic with SARS-CoV-2 is of paramount importance for public health in order to assess the potential benefit of immunity and design future preventive interventions.

To our knowledge, this is the first study in Africa to assess the long-term durability and longitudinal profile of anti-SARS-CoV-2 antibody levels in a large cohort of seropositive- patients beyond 12 months. This study aims to assess the kinetics of IgG, IgM and IgA antibodies up to 12 months post-infection in a large cohort of COVID-19 patients in Africa, and to analyze the impact of host factors such as age, sex and comorbidities on antibody levels during this period.

## Materials and methods

### Study design and participants

The sample size for this study was established using a sample size calculator for proportions, with the following assumptions: a COVID-19 positivity rate of 20% in the Moroccan population at the time of study initiation, a desired margin of error of 5% and a desired statistical power of 80%. Based on these assumptions, the calculated sample size needed for the study was approximately 246 individuals. However, we did not reach this number of patients because vaccination in Morocco began on January 29, 2021, targeting the elderly population and health personnel. We therefore had difficulty recruiting elderly and young people who usually develop only asymptomatic or mild forms of the disease. A total of 190 symptomatic patients (within 5 days of symptom onset) who tested positive for COVID-19 using quantitative reverse transcription polymerase chain reaction (RT-qPCR) between 18 March and 8 June 2021 were enrolled in the study at two hospitals in Casablanca (Moulay Youssef regional hospital and Mohamed Bouafi hospital). Sample collection at different follow-up time points was performed between March 25, 2021 and June 10, 2022. Their antibody status was assessed at different follow-up time points (day 00 to day 360 after symptom onset) between 15 July and 30 December, 2022. Clinical characteristics, such as gender, age, comorbidities, SARS-CoV-2 vaccination status, and disease information (including the date of first symptoms and clinical signs), were collected for each patient. The study included adult men and women who were either unvaccinated (167 patients) or had received the ChAdOx1 nCoV-19 vaccine before infection (23 patients). The corresponding author has access to all information provided by participants.

### Ethics statement

All participants gave written informed consent. Study procedures were conducted in accordance with the 1964 Declaration of Helsinki. The study protocol was approved by the Ethics Committee of the Mohammed VI University of Health Sciences in Casablanca. All donor data were anonymized after inclusion in the study.

### Sample collection

Venous blood was collected in dry tubes and centrifuged at 900g for 10 minutes. Serum supernatants were stored at -20°C. Serum samples were collected from COVID-19 patients at various time points after symptom onset, including 2–5 days (D00), 9–12 days (D07), 17–20 days (D15), 32–37 days (D30), 62–67 days (D60), 92–98 days (D90), 182–188 days (D180), and 363–367 days (D360). Participants were separated into two groups: one group consisted of infected and unvaccinated patients over a 12-month period, while the other group consisted of patients who had been vaccinated with ChAdOx1 nCoV-19 (AstraZeneca) before infection. Patients who received mRNA or vector vaccines (Pfizer, AstraZeneca, Johnson) during follow-up were excluded from the anti-RBD IgG antibody analysis, and patients who received BBIBP-CorV (Sinopharm) were excluded from all analyses. Furthermore, throughout the study, some patients were excluded for other reasons, such as relocation from the city, hospitalization and unavailability due to work. Samples from patients reinfected with SARS-CoV-2 between study visits were not included.

### Detection of anti-SARS-CoV-2 antibodies

We detected anti-SARS-CoV-2 IgG, IgM, and IgA antibodies against receptor-binding domain of S protein, and the N using three serological techniques: anti-N IgA and IgG were measured using an in-house ELISA. This ELISA was developed in Tunisia and its performance was tested in different African settings with variable endemicity. In fact, we participated in the evaluation and validation of this in-house ELISA [34]. The sensitivity of the anti-N IgG ELISA test was 92% and specificity 94%. The sensitivity and specificity of the anti-N IgA ELISA test were 87% and 95%, respectively.

To assess the presence of anti-N IgM antibodies in human sera, a semi-quantitative ELISA (Euroimmun ELISA) was used. The commercial anti-SARS-CoV-2 nucleocapsid protein-specific IgM ELISA kit (Euroimmun, Lübeck, Germany) was used, with a sensitivity and specificity of 88.2% and 98.6%, respectively. The manufacturer’s instructions were followed for analysis and interpretation of results. Evaluation consisted of calculating the ratio of control or patient sample extinction to calibrator extinction. A ratio <0.8 was interpreted as negative, ≥0.8 to <1.0 as borderline, and ≥1.1 as positive. Borderline results were considered negative for the analysis.

To quantify anti-RBD IgG antibody, samples from unvaccinated patients were tested using the Abbott SARS-CoV-2 IgG II Quant assay on the Abbott ARCHITECT i2000SR immunoassay instrument (Abbott Laboratories, Abbott Park, Illinois). This automated platform has a high sensitivity of 98.3% and specificity of 99.5% for antibody detection. The manufacturer has defined the analytical measurement range to be between 21 and 40,000 AU/mL, with a cutoff point of ≥50 AU/mL. A protective threshold value of ≥4000 AU/mL has been established for the Abbott Architect test [35]. This assay showed high agreement with neutralizing antibody titers [36] and is capable of detecting antibodies in individuals infected with two different variants of concern, namely the VOC 202012/V1 [UK] and VOC 202012/V2 [South Africa] strains [37].

### Statistical analysis

Categorical variables were reported as proportions and numbers, while continuous variables were described using the median and interquartile range (IQR) for the descriptive statistics. The Mann-Whitney test was used to compare continuous variables between groups. All statistical analyses were performed using R software package (https://www.r-project.org), and a significance level of 0.05 was used. Statistical tests were two-sided.

## Results

### Clinical and demographic characteristics of enrolled patients at baseline

In our study, we collected data from 190 participants whose SARS-CoV-2 infection was confirmed by a positive RT-qPCR test. Participants had a median age of 47.5 years, ranging from 18 to 77 years, and most of them were females (61.6%). More than half of the participants were adults younger than 50 years (55.8%). The most common symptoms reported by participants were muscle pain (71.6%), cough (64.2%), headache (61.1%), and chills (54.2%). Other symptoms included fever (46.3%), loss of appetite (39.5%), and sore throat (37.9%). Of all patients, 34% had at least one preexisting comorbidity, with diabetes being the most common comorbidity (22 patients). These demographic and clinical data are presented in Table 1.

**Table 1:** Clinical and demographic characteristics of the study cohort.

Characteristics	n (%)
Total	190 (100)
Gender	
Female	117 (61.6)
Male	73 (38.4)
Age (median [IQR])	47.5 [30.0–57.7]
Age (year)	
18–35	63 (33.2)
35–50	43 (22.6)
>50	84 (44.2)
Comorbidities*	34 (17.9)
Daibetes	22
DiabetesAsthma	8
Cardiovascular disease	5
Obesity	4
Symptoms	
Muscle pain	136 (71.6)
Cough	122 (64.2)
Headache	116 (61.1)
Chill	103 (54.2)
Fever	88 (46.3)
Loss of appetite	75 (39.5)
Sore throat	72 (37.9)
Asthenia	68 (35.8)
Diarrhea	56 (29.5)
Nausea	48 (25.3)
Dyspnea	43 (22.6)
Abdominal pain	37 (19.5)
Vomiting	21 (11.1)

* Number and percentage of patients with at least one comorbidity.

### Natural history of humoral response up to one year after COVID-19

**Fig 1:**
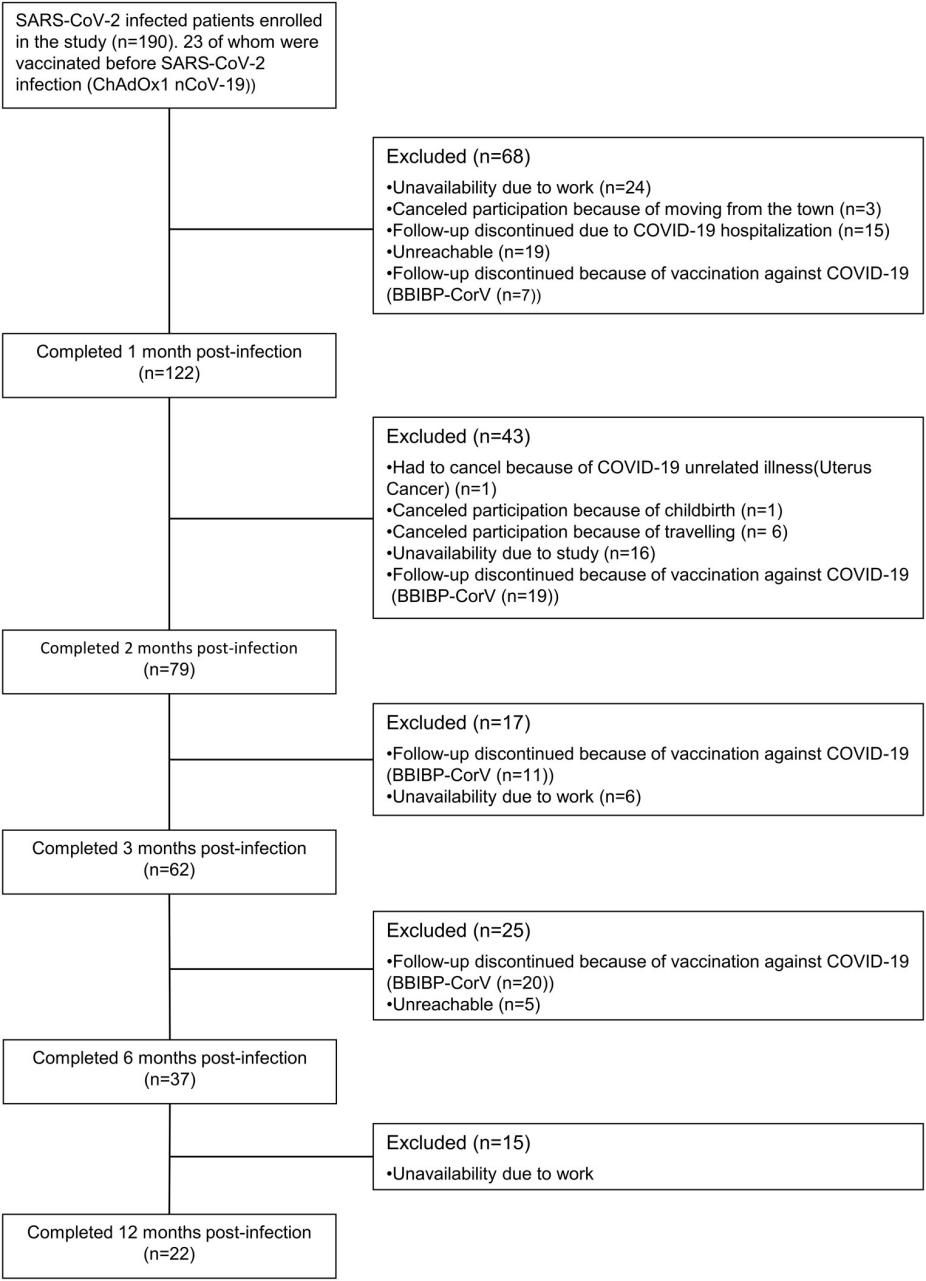
Study flowchart. BBIBP-CorV: Sinopharm vaccine; ChAdOx1 nCoV-19: AstraZeneca; COVID-19 vaccine: Coronavirus disease 2019; SARS-CoV-2: Severe acute respiratory syndrome coronavirus 2.

In this study, IgM, IgA, and IgG antibody kinetics were assessed in one hundred ninety patients diagnosed with SARS-CoV-2 infection by RT-PCR. These patients were followed for one year. Throughout the study, some patients were excluded for various reasons, such as relocation from the city, hospitalization, unavailability due to work or vaccination, as illustrated in Fig 1.

### SARS-CoV-2 during a one-month follow-up

**Fig 2:**
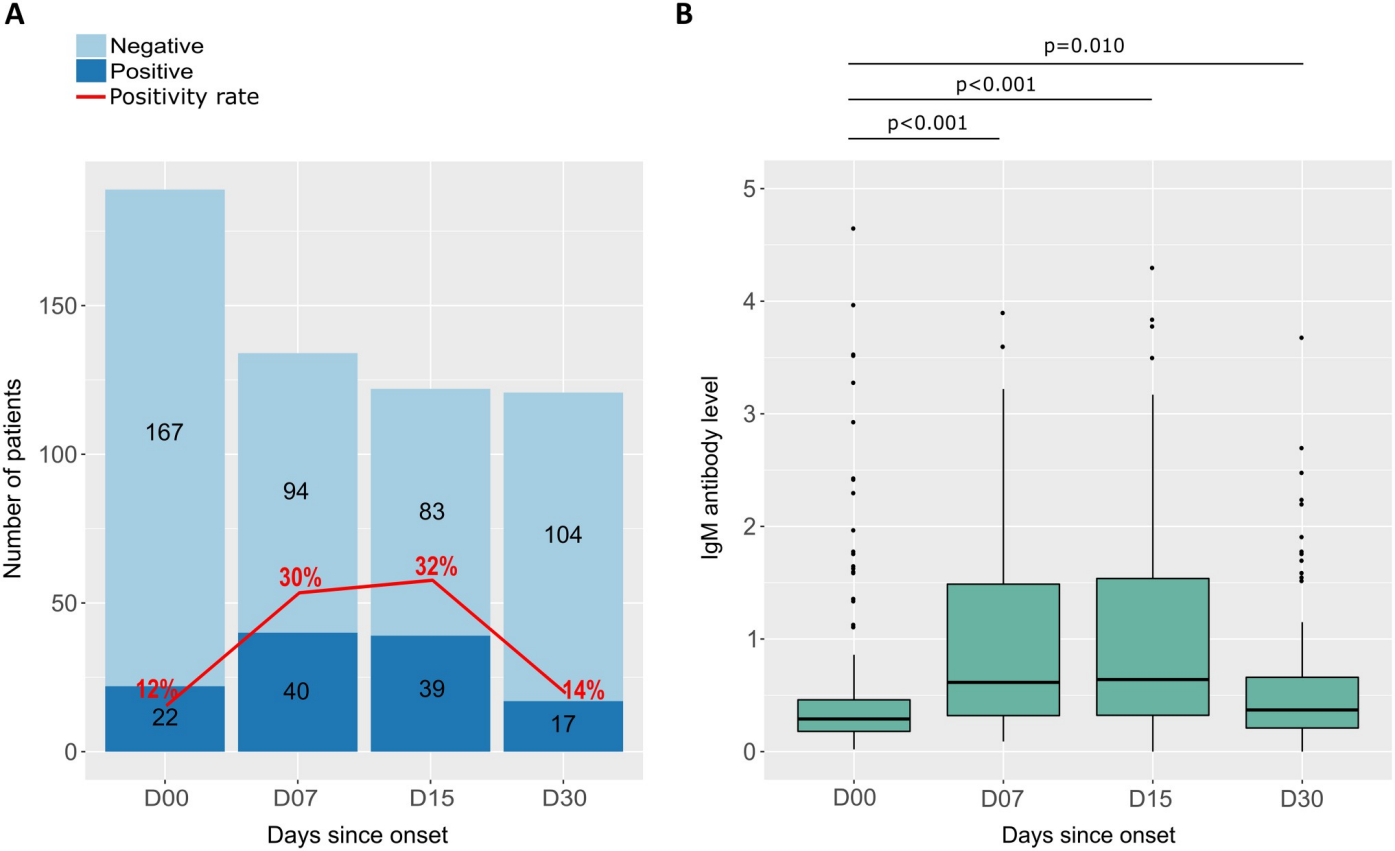
Seropositivity rates and IgM antibody levels in response to SARS-CoV-2 as a function of time since symptom onset. (A) The graph shows the number of participants tested positive (dark blue) and negative (light blue) for IgM antibodies, as well as the rate of seropositivity (red curve) as a function of the number of days after symptom onset at follow-up. (B) Box plots s display the distribution and differences in anti-SARS-CoV-2 IgM antibody levels for participants sampled at different follow-up periods. P-values were calculated using the Mann-Whitney test.

IgM antibodies were measured in the serum of the COVID-19 patient for one month after the onset of symptoms. The follow-up period was divided into four time intervals: D00 (2–5 days after symptom onset), D07, D15, and D30. In the first 20 days after symptom onset (between J00 and J15), the IgM seropositivity rate fluctuated between 12% and 32% and then decreased to 14% at 1 month after symptom onset (Fig 2A). At the same time, the median IgM antibody level was initially 0.29 (IQR 0.18–0.46) and increased at 1 to 2 weeks after symptom onset to 0.64 (IQR 0.32–1.54), and then decreased to 0.37 (IQR 0.21–0.66) at 1 month after symptom onset. Differences in median antibody levels between D00 and D07, between D00 and D15 and between D00 and D30 were statistically significant (Fig 2B).

**Fig 3:**
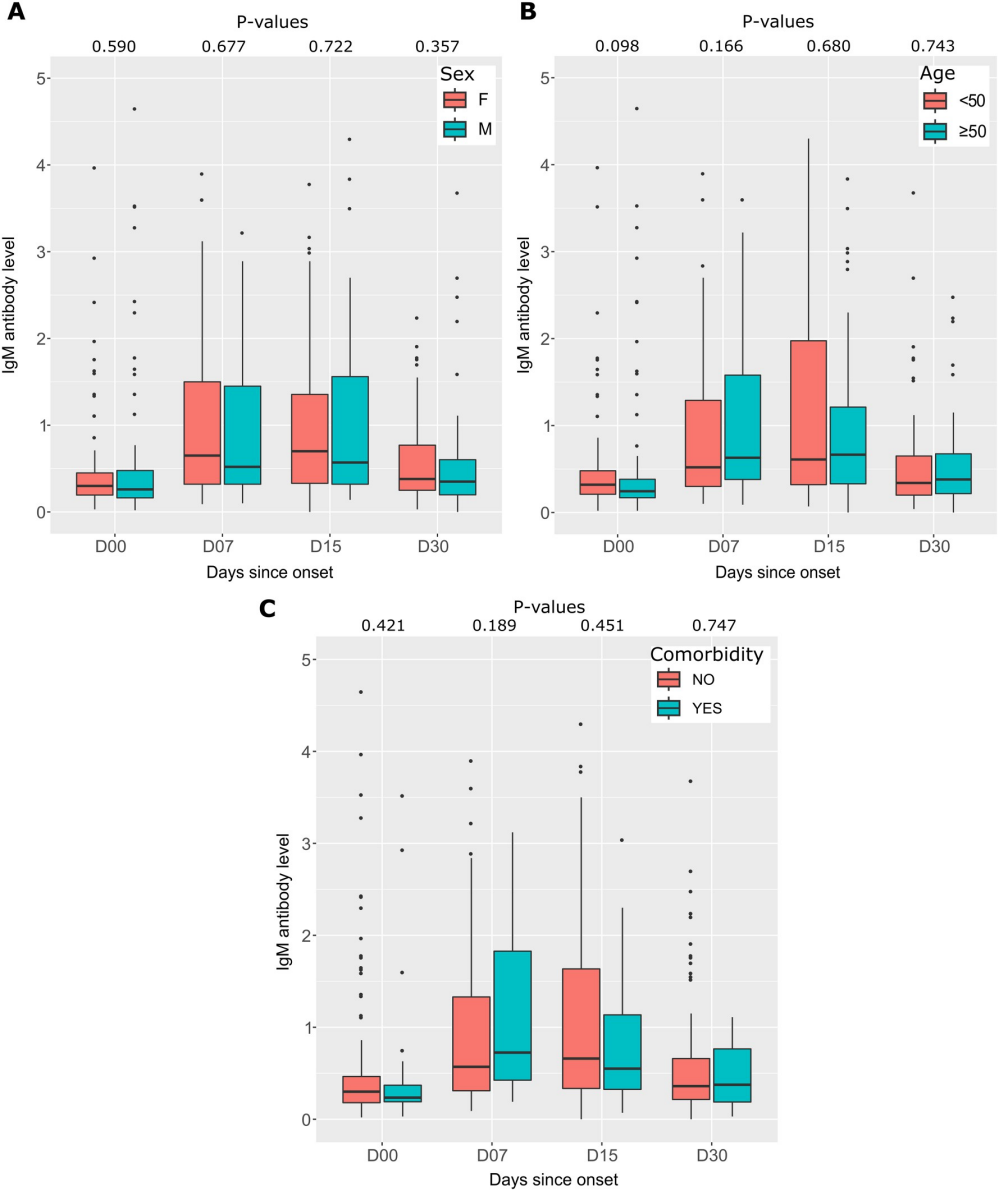
The differences in IgM antibody levels between different groups. (A) The first graph compares IgM antibody levels between genders (F for female and M for male), showing the distribution of antibody levels in each group. (B) IgM antibody levels are shown as a function of age, with the 190 participants divided into two groups: under 50 years and over 50 years. Each age interval is represented by a separate box plot. (C) IgM antibody levels according to the presence or absence of comorbidities. The box plots show the distribution of antibody levels in each group. In all three graphs, the P-values are calculated using the Mann-Whitney test.

Furthermore, IgM response was evaluated by gender, age, and comorbidities (Fig 3). The median IgM antibody level was almost similar between women and men, and there was no significant difference over the four follow-up time intervals (Fig 3A). Participants were aged 18 to 67 years and were divided into two groups: group 1 (<50) and group 2 (⩾50). There was no significant difference in the median IgM antibody level according to the age of the participants (Fig 3B).

Patients were classified according to the presence or absence of preexisting illnesses. Patients with comorbidities had higher median antibody levels 1 week after symptom onset (0.73 [IQR 0.43–1.83] versus 0.57 [IQR 0.31–1.33]), respectively. However, this difference was not significant (p = 0.189), and median antibody levels were similar in both patient groups at the other follow-up time intervals (Fig 3C).

### IgA responses to SARS-CoV-2 during one-month follow-up

**Fig 4:**
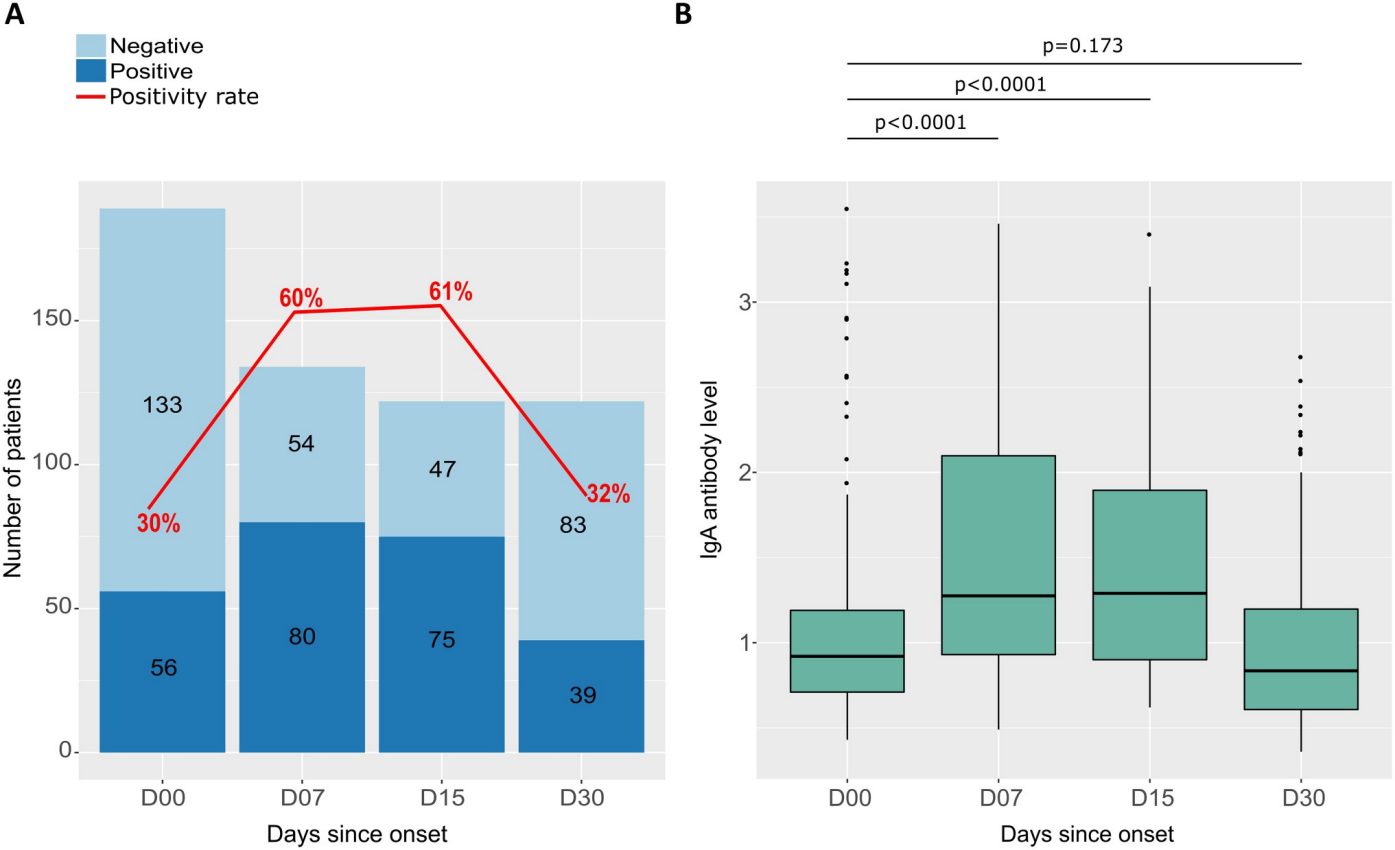
Seropositivity rates and IgA antibody levels in response to SARS-CoV-2 as a function of time since symptom onset. (A) Graph shows the number of participants tested positive (dark blue) and negative (light blue) for IgA antibodies, as well as the rate of seropositivity (red curve) as a function of the number of days after symptom onset at follow-up. (B) Box plots show the distribution and differences anti-SARS-CoV-2 IgA antibody levels for participants sampled at different follow-up periods. P-values were calculated using the Mann-Whitney test.

As with IgM, IgA antibodies were measured in the serum of COVID-19 patients over the same follow-up periods as for IgM. IgA antibody kinetics was similar to those of IgM, but IgA positivity rates were higher than those of IgM. It was 30% at D00, increased to 60% at 1 week after symptom onset, and remained stable at 2 weeks after symptom onset (61%), after which the antibody level decreases to 32% (Fig 4A). Meanwhile, the median IgA antibody level was initially 0.92 (IQR 0.71–1.19) and increased 1–2 weeks after symptom onset to 1.29 (IQR 0.90–1.90), then the IgM level decreased to 0.84 (IQR 0.61–1.20) 1 month after symptom onset. Differences in median antibody levels between D00 and D07 and between D00 and D15 were statistically significant (p <0.0001). The median antibody level decreased to almost the same value as at baseline one month after symptom onset (p = 0.173) (Fig 4B).

**Fig 5:**
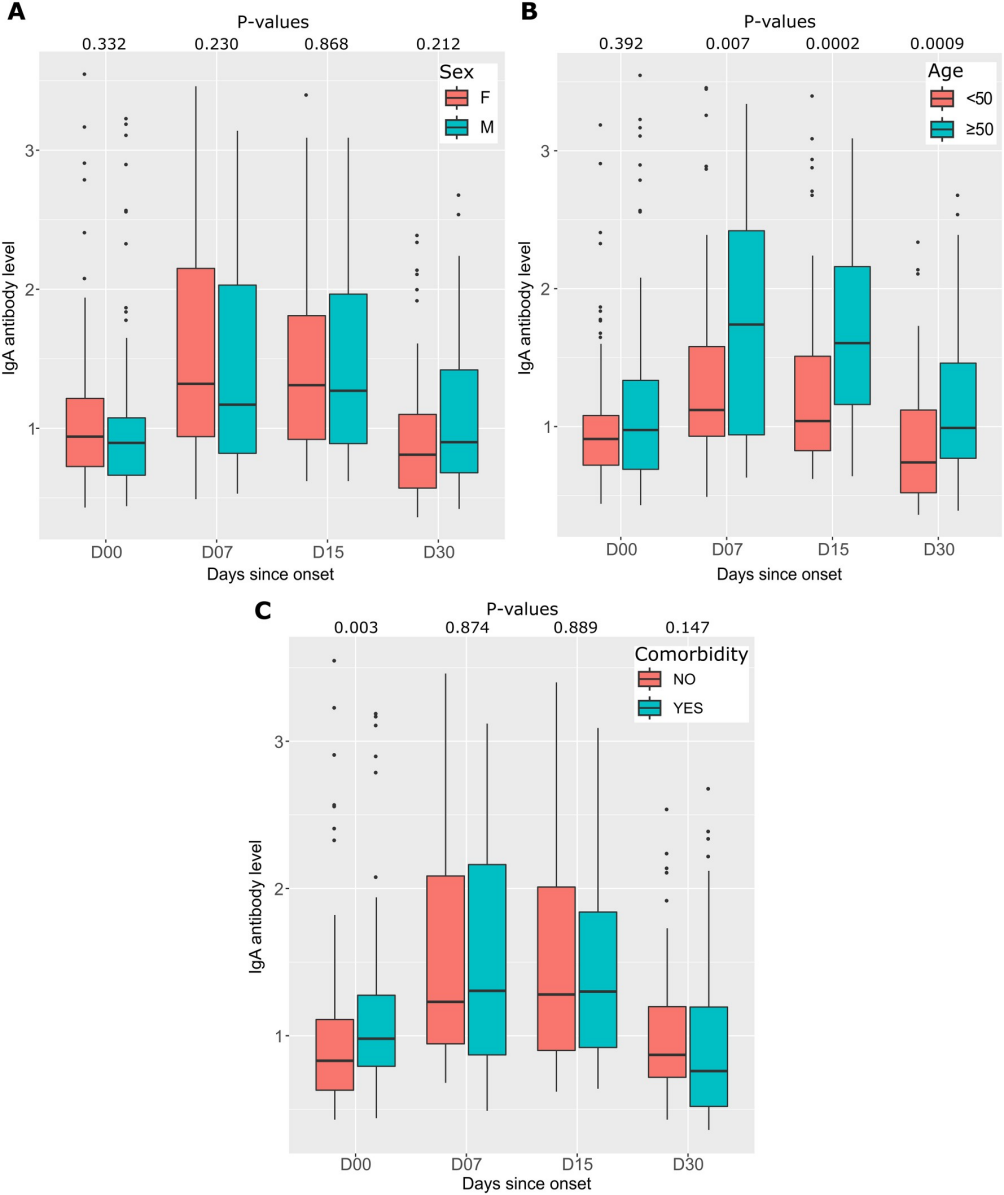
Differences in IgA antibody levels between different groups. (A) The first graph compares the IgA antibody levels between the sexes (F for female and M for male), showing the distribution of antibody levels in each group. (B) IgA antibody levels are shown as a function of age, with the 190 participants divided into two groups: under 50 years and over 50 years. Each age interval is represented by a separate box plot. (C) Displays IgA antibody levels according to the presence or absence of comorbidities. The box plots show the distribution of antibody levels in each group. In all three graphs, P-values are calculated using the Mann-Whitney test.

We observed an increase in IgA antibody levels in in the over-50-year-old group of patients compared with the under-50-year-old group (Fig 5B). However, as with IgM antibodies, there were no significant differences in median levels between genders or comorbidities, except at the onset of infection (D00) when there was an increase in antibody levels in patients with comorbidities (0.98 (IQR 0.79–1.14) versus 0.83 (IQR 0.63–1.11), respectively (p = 0.003)) (Fig 5C).

### SARS-CoV-2 anti-RBD IgG responses during a 12-month follow-up

**Fig 6:**
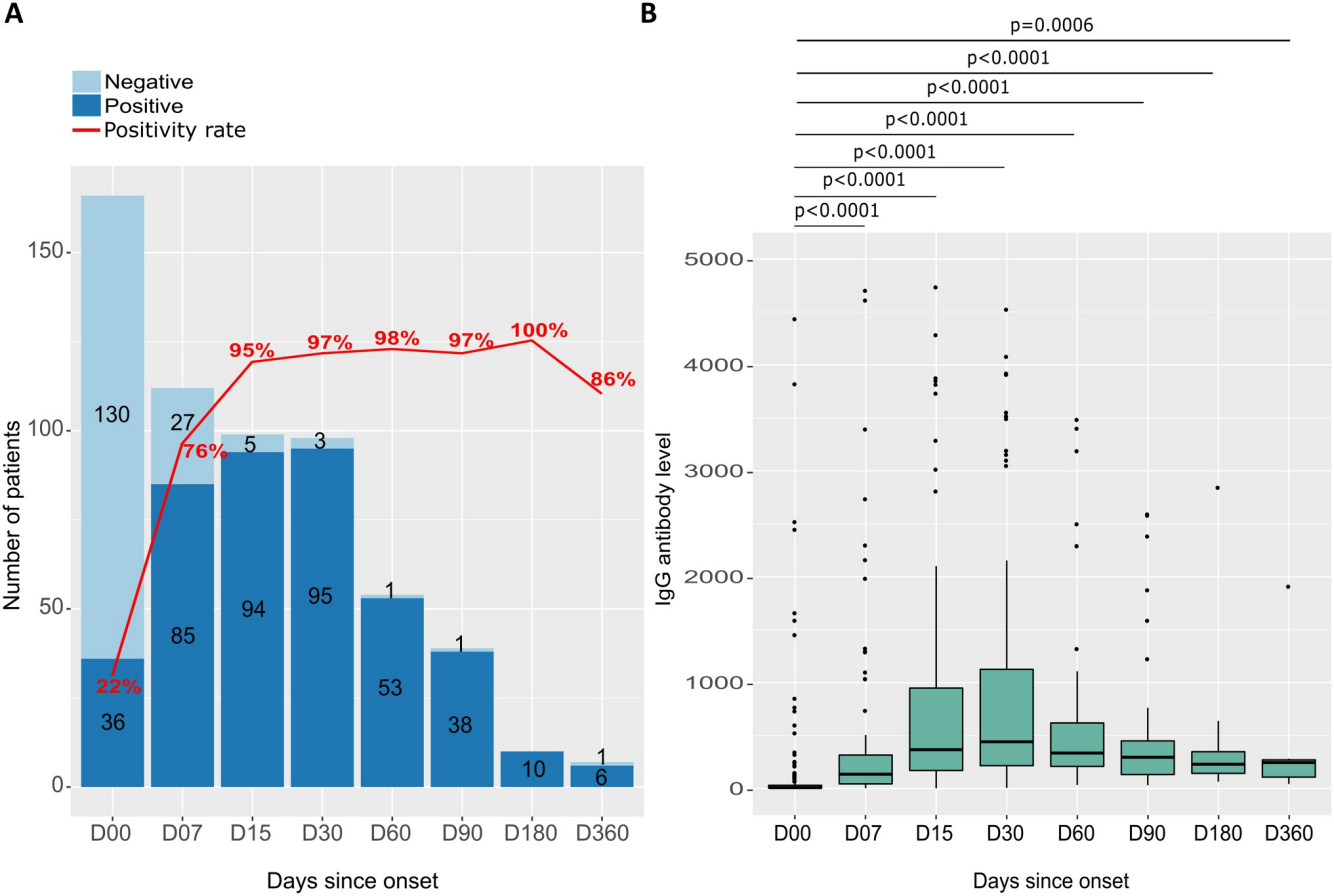
Seropositivity rate and anti-RBD IgG antibody level in response to SARS-CoV-2 as a function of time since symptom onset. (A) The number of participants ested positive (dark blue) and negative (light blue) for anti-RBD IgG antibodies, as well as the seropositivity rate (red curve) as a function of days after symptom onset at follow-up. (B) Distribution and difference in levels of anti-SARS-CoV-2 anti-RBD IgG antibody levels for participants sampled at different follow-up periods. Each box plot represents a different follow-up period, and P-values are calculated using the Mann-Whitney test.

During the 12-month follow-up period, eight different recruitment periods were used (ranging from 2 to 5 days after symptom onset to 12 months post-onset), denoted as D00, D07, D15, D30, D60, D90, D180, and D360. During this period, the proportion of individuals testing positive for anti-RBD IgG antibodies increased steadily from 22% at D00 to 95% at 2 weeks after symptom onset. Between D15 and D180, the seropositivity rate remained relatively stable, fluctuating between approximately 95% and 98%, with a peak of 100% at six months. Whereas, at 12 months after symptom onset, there was a slight decrease in the proportion of individuals testing positive for IgG antibodies, with a seropositivity rate of 86% (as shown in Fig 6A). Median anti-RBD IgG levels increased over time to a maximum 30 days after symptom onset. It increased from 8.50 [IQR 4.90–30.80] to 476.80 [IQR 234.50–647] from D00 to D30, respectively (Fig 6B) and reached 245.60 [IQR 108.20–270.90] at 1 year after symptom onset.

**Fig 7:**
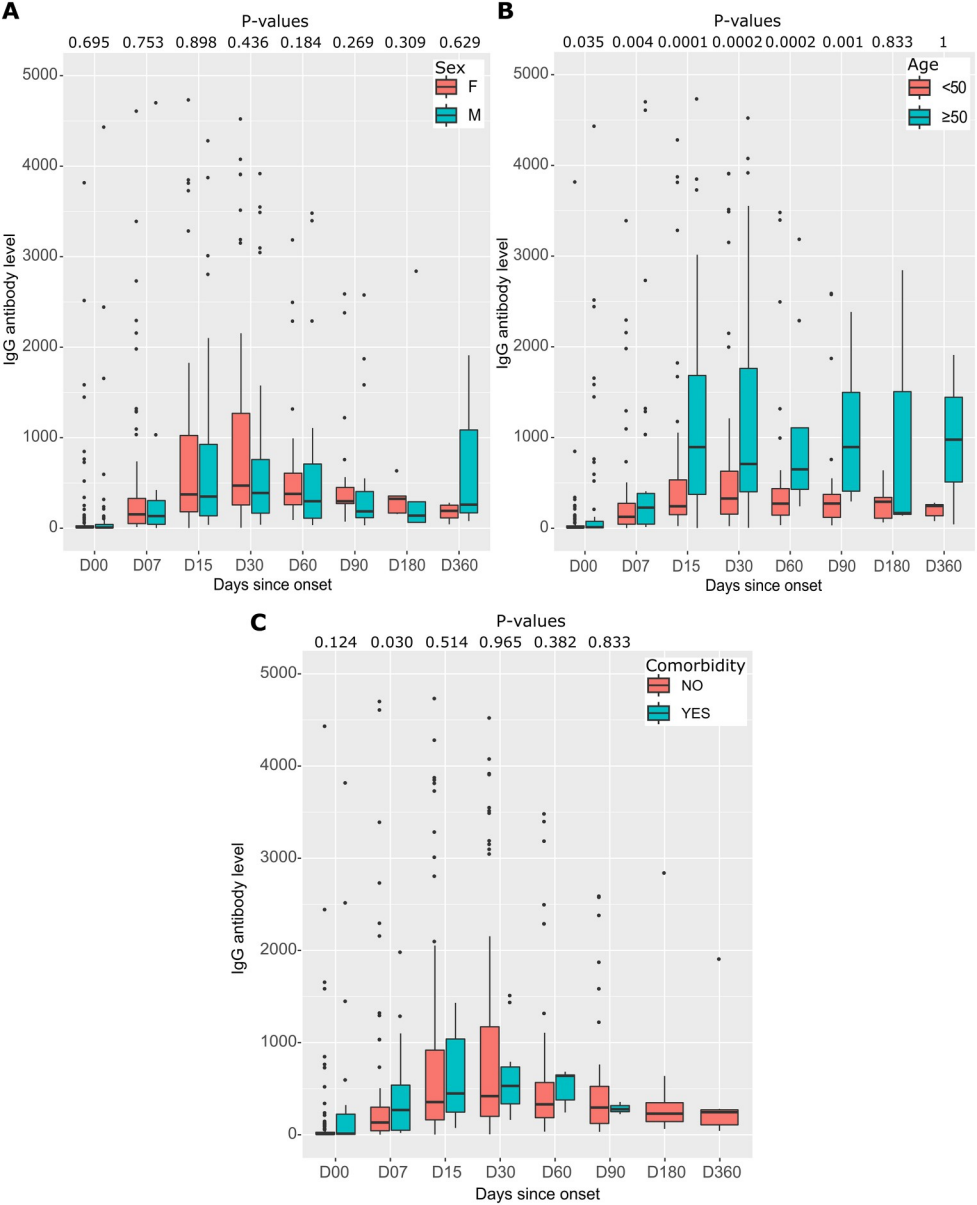
Distribution and differences of anti-RBD IgG antibodies to SARS-CoV-2 by sex, age, and comorbidity during follow-up. (A) anti-RBD IgG antibody levels by sex, with the distribution of antibody levels in each group represented by box plots. (B) anti-RBD IgG antibody levels by age, with the 167 participants divided into two groups based on age: under 50 years and over 50 years. Each age range is represented by a separate box plot. (C) IgG antibody levels according to the presence or absence of comorbidities, with the distribution of antibody levels in each group represented by box plots. In all three graphs, P-values are calculated using the Mann-Whitney test.

Median IgG antibody levels were almost similar in women and men at different follow-up times (Fig 7A). Patients over 50 years of age had very high median IgG antibody levels compared with those under 50. This difference was statistically significant from D00 to D90 (p < 0.05) (Fig 7B). Participants with comorbidities had higher median IgG antibody levels than those without pre-existing pathologies, but the differences were not statistically significant (Fig 7C).

### SARS-CoV-2 IgG anti-N responses during a 12-month follow-up

**Fig 8:**
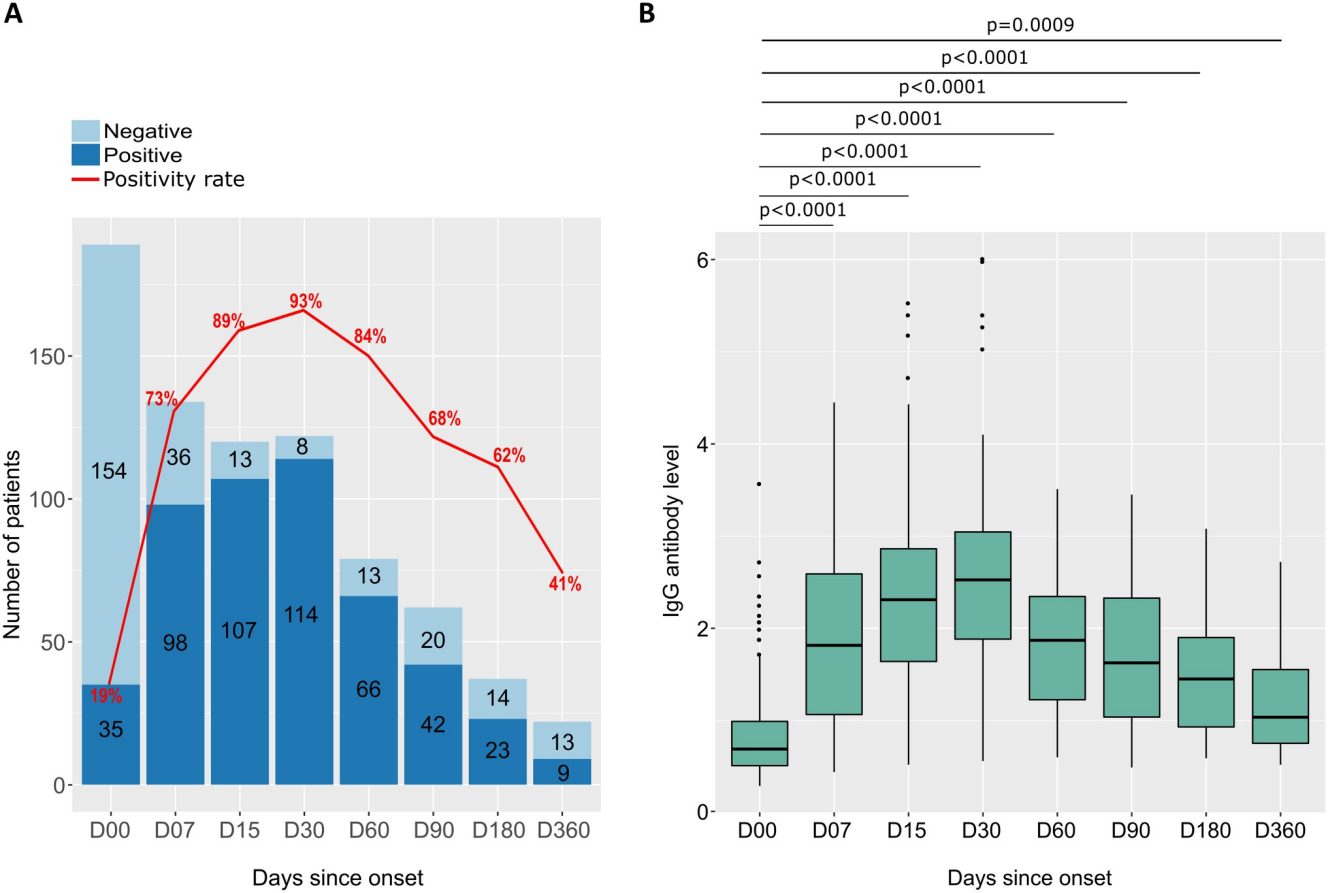
Seropositivity rate and anti-N IgG antibody levels in response to SARS-CoV-2 as a function of time since symptom onset. (A) The number of participants tested positive (dark blue) and negative (light blue) for anti-N IgG antibodies, as well as the seropositivity rate (red curve) as a function of the number of days after symptom onset at follow-up. (B) Distribution and difference in levels of of anti-SARS-CoV-2 anti-N IgG antibody levels for participants sampled at different follow-up periods. Each box plot represents a different follow-up period, and P-values are calculated using the Mann-Whitney test.

As with anti-RBD IgG, anti-N IgG was measured in patient serum during the 12-month follow-up period at eight different time points during recruitment. The seropositivity rate for anti-N IgG increased from 19% at D00 to a peak at 1 month after symptom onset (93%). Thereafter, the anti-N antibody level decreased to 41% at 1 year after symptom onset (Fig 8A). Median IgG levels increased over time, peaking at D30. Thereafter, median IgG levels decreased with time until D360, but remained higher than the baseline at D00 (Fig 8B).

**Fig 9:**
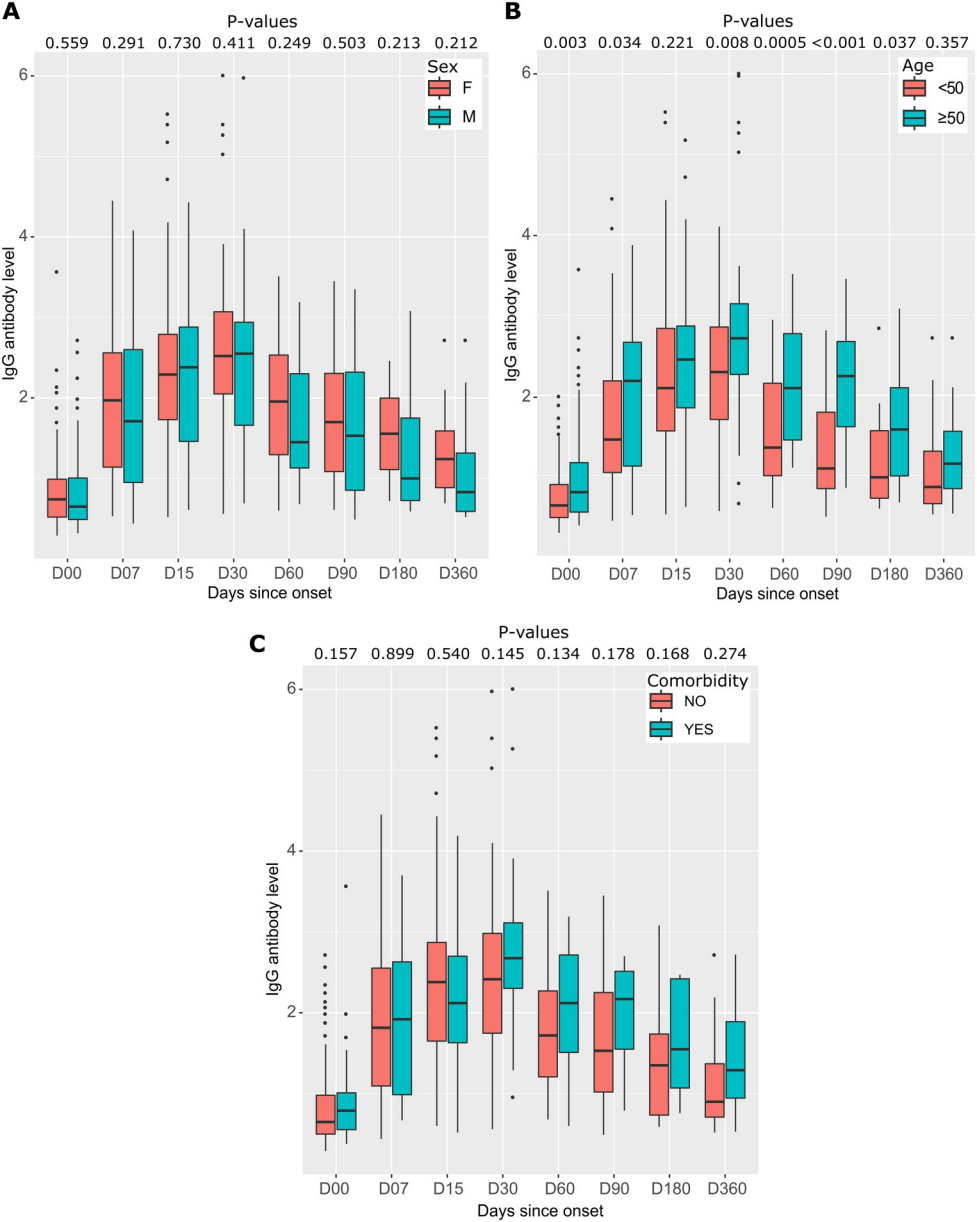
Distributions and differences of anti-N IgG antibodies to SARS-CoV-2 by sex, age, and comorbidity at follow-up. (A) anti-N IgG antibody levels by sex, with the distribution of antibody levels in each group represented by box plots. (B) anti-N IgG antibody levels by age, with the 190 participants divided into two groups based on age: under 50 years and over 50 years. Each age range is represented by a separate box plot. (C) anti-N IgG antibody levels according to the presence or absence of comorbidities, with the distribution of antibody levels in each group represented by boxplots. In all three graphs, P-values are calculated using the Mann-Whitney test.

Antibody levels were assessed in men and women at different time points after symptom onset. Although median IgG antibody levels were slightly higher in women than men at 1 week, 6 months, and 12 months, these differences were not statistically significant (as shown in the Fig 9A). In addition, the study found that patients aged 50 years and older had significantly higher median IgG antibody levels than those younger than 50 years at different time points, with the highest peak at Day 30 (2.71 [IQR 2.26–3.14] versus 2.29 [IQR 1.70–2.85], respectively), with a P-value of 0.008 (as shown in Fig 9B). Furthermore, the study also revealed that participants with pre-existing pathologies had higher median IgG antibody levels over time than those without such pathologies, but that these differences were not statistically significant (as shown in Fig 9C).

### Impact of SARS-CoV-2 vaccination on humoral responses in COVID-19 positive patients

**Fig 10:**
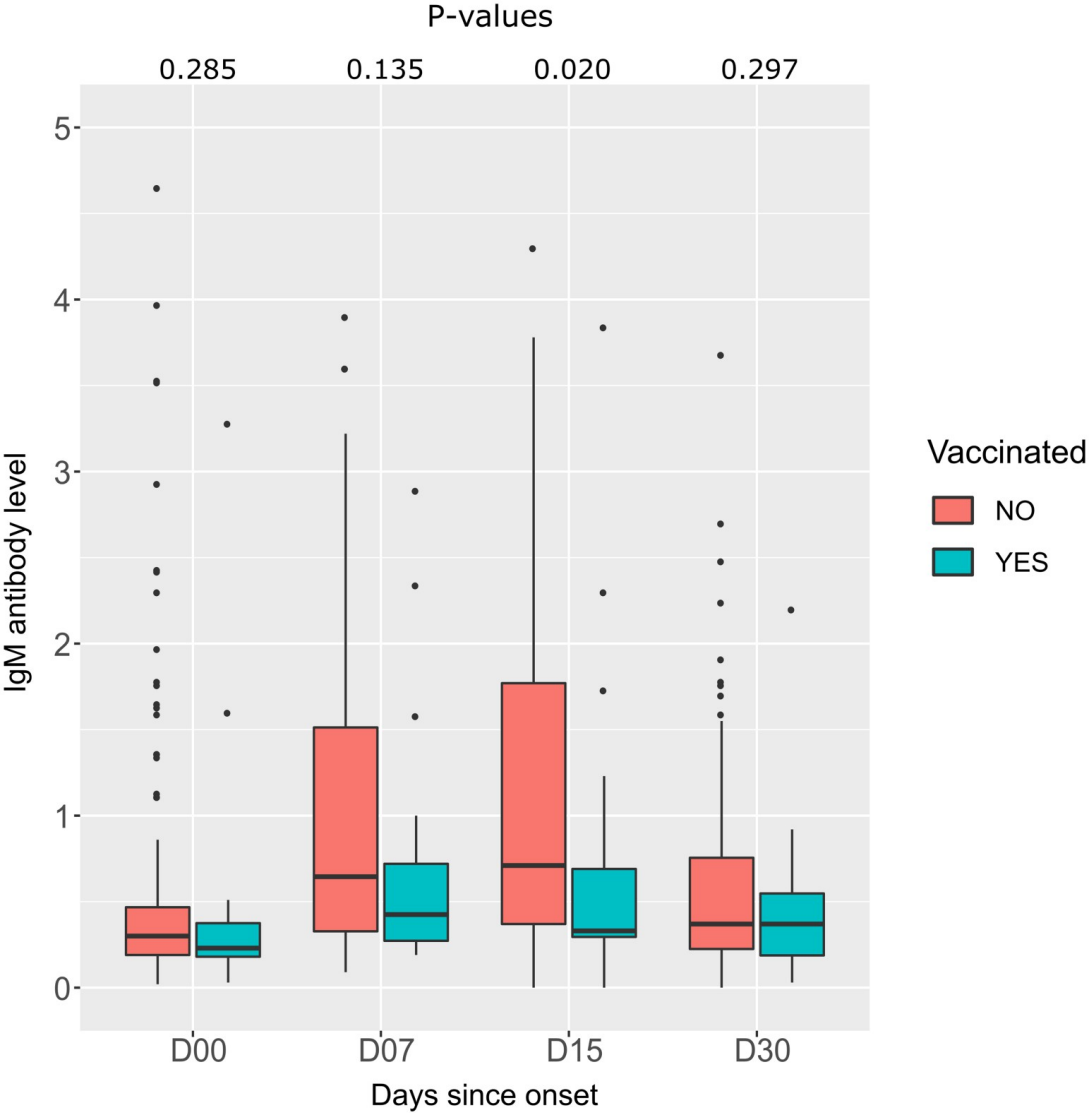
Comparison of IgM antibody levels between two groups of patients according to time since onset of symptoms. Unvaccinated infected patients (red box) and vaccinated patients before infection (green box). P-values are calculated by the Mann-Whitney test.

We analyzed the antibody level in two groups of patients: patients vaccinated with ChAdOx1 nCoV-19 before infection and unvaccinated patients. The median IgM level was lower in the vaccinated group than in the unvaccinated group. This difference is statistically significant at 2 weeks after the onset of symptoms (p = 0.020) (Fig 10).

## Discussion

Blood IgA, IgM, and IgG immunoglobulins determinations are essential to understand the mechanisms of systemic humoral immune responses against SARS-CoV-2 in patients who have tested positive for COVID-19 using PCR. The study of the dynamics of these antibodies is essential for diagnosis as well as for studying sensitivity or resistance to subsequent reinfection [38]. In general, IgA and IgM antibodies are generated earlier than IgG isotypes, with earlier detection of IgA than IgM and remarkable persistence of IgG over time [39–41]. We previously described the kinetics of anti-N IgM and anti-S IgG antibody responses up to 3 months after natural infection using a commercial Euroimmun ELISA [42]. In this study, we used several serological assays to measure IgA and IgM antibodies levels against the nucleocapsid protein (NCP) at 1 month after symptom onset, and monitored IgG against the RBD in the S1 subunit and anti-N IgG up to 1 year after infection. We also analyzed the effect of age, gender, and comorbidities on the evolution of antibody responses over time. Our findings indicate that IgA and IgM antibody responses exhibit similar kinetics during the first month after SARS-CoV-2 infection, but the intensity of the IgA response is greater than that of the IgM response. Additionally, IgG anti- RBD antibody levels peaked at 1 month after symptom onset and remained elevated up to one year of follow-up, while IgG anti-N antibody levels decreased over time. The N protein is an intracellular protein released during viral replication. A high level of anti-N antibodies could indicate a high level of virus replication and consequently a high viral load [43]. In asymptomatic forms, the level of anti-N antibodies is low [44,45]. It has been observed that the anti-N antibody level is lower than the anti-RBD antibody level in asympromatic patients, and vice versa in deceased patients, where an increase in the anti-N antibody level is observed. This could be explained by:i) the different forms in which these proteins exist in vivo, as well as cross-binding of B cell receptors (BCRs) or dual signaling by BCRs and Toll-like receptors (TLRs). These factors may contribute to the differential activation and proliferation of protein-specific B cells, leading to differences in the quantity and persistence of the resulting antibody response [46,47].ii) differences in the activation of long-lived plasma cells by surface antigens (RBD) or internal antigens (N). iii) by increased avidity or affinity that compensates for antibody loss, or by changes in the epitopes recognized over time [48]. Two weeks after symptom onset, 32% of patients had detectable SARS-CoV-2-specific IgM antibodies, while 61% were seropositive for IgA. Previous studies have highlighted the differences between IgM and IgA antibody titers in response to SARS-CoV-2 infection. The duration of persistence of anti-SARS-CoV-2 antibodies after infection is an important clinical issue. Whereas, some studies suggest that the humoral response may persist for several months [24,49], others indicate a decrease in anti-SARS-CoV-2 antibodies during the first few months [21,22,50]. Anti-RBD antibodies have been shown to persist for a prolonged period, potentially reducing the risk of reinfection in people previously infected with SARS-CoV-2 [48]. In addition, reinfection has been reported in approximately 1% of patients [51–54], highlighting the importance of understanding the longevity of the immune response to SARS-CoV-2. IgA antibodies were of particular interest due to their presence in mucosal surfaces, such as the respiratory and gastrointestinal tracts, where SARS-CoV-2 is primarily infected. The results of the study showed that IgA antibodies were highly present in the samples collected, particularly during the initial phase of SARS-CoV-2 infection. This suggests that the immune system rapidly produces IgA antibodies in response to the virus. In addition, the study revealed that serum-derived IgA and IgA antibodies from mucosal surfaces play a more important role in virus neutralization than IgG antibodies [55]. A study of patients with confirmed infection by RT-qPCR showed that IgA responses were detected earlier and peaked at week 3, with a stronger and more persistent response than IgM [39]. Another study by Carnicelli et al. reported that IgA was detectable as early as 5 to 7 days after symptom onset, peaked between days 21 to 27, and remained stable thereafter, with a declining trend observed after day 50 [56]. A study of 58 COVID-19 patients showed that IgA titers generated against N increased more rapidly than IgM titers up to day 10. After day 45, IgA titers N began to decline. However, IgG titres remained high, and were the highest of the three antibody types studied. The study showed that high IgG titers (RBD) were maintained for more than 6 months [57]. In a different study, 233 convalescent individuals were followed for 300 days. The study found that the peak response for COVID-19 patients was between 16 and 30 days for IgG, 15 to 22 days for IgM, and 0 to 60 days for IgA. The levels of these antibodies gradually decreased after peaking, but were still detectable up to 300 days, with the exception of IgM antibodies which disappeared between 61 and 90 days in all patients [58]. It has been described that IgA antibodies, present in the serum, saliva, and bronchoalveolar washings of patients, are more effective in neutralizing virus than IgG antibodies [59]. Furthermore, IgA dimers, present in mucosal surfaces, are even more effective in neutralizing viruses than IgA monomers found in serum. This indicates that IgA antibodies may play a crucial role in the prevention of viral infections, their transmission and the worsening of symptoms. Our study shows that IgG antibody levels peak one month after the onset of symptoms, with 97% and 93% seropositivity for anti-RBD IgG and anti-N IgG, respectively. Anti-RBD IgG levels remained elevated through 1 year of follow-up. In contrast, anti-N IgG antibody levels decreased over time, with only 41% of patients testing positive after 1 year of follow-up. Several studies have confirmed the persistence of IgG antibodies, a study of 256 healthcare workers who tested positive for COVID-19 by RT-qPCR showed that IgG seropositivity peaked between 21 and 28 days after the onset of symptoms. Thereafter, anti-N IgG began to decrease after one month of infection. However, anti-S IgG persisted up to 7 months in 93.4% of cases [60]. Another study reported that the half-life of S-RBD-targeting IgG was 126 days, whereas N-specific IgG decayed much more rapidly, with a half-life of 71 days [61]. A study in COVID-19 transplant patients reported a disparity between anti-N and anti-RBD IgG antibody levels, with anti-N antibody levels being lower than anti-RBD IgG [62]. IgG increases rapidly from the first month and fluctuated between the third and sixth month, with a gradual decrease over the following months [63]. Choteau et al. showed that peak IgG seropositivity was reached between 10 and 15 days after the onset of symptoms, with most patients still having anti-SARS-CoV-2 IgG (88.68%). However, anti-RBD IgG and anti-N IgG tended to decrease between days 61 and 105 after symptom onset [55]. In contrast, another study reported that the positivity rate of samples collected between 180 and 330 days after symptom onset reached 100% for anti-RBD IgG and 96.68% for anti-N IgG [64]. The differences observed in the results of these studies could be explained by several factors, including the size of the cohort, the types of serological tests used (with their respective sensitivity and specificity) and the patient population studied. In addition, the clinical results of the COVID-19 study may also contribute to the observed differences in IgG antibody persistence. On the basis of our analysis, we investigated whether the diverse antibody responses observed in patients who recovered from COVID-19 could be related to disparities in patient gender, age, or comorbidities. Our study, similar to previous ones [65–68], found that there were no significant variations in median anti-N and/or anti-RBD antibody levels in infected patients based on gender or comorbidities. In contrast, Korte et al observed gender discrepancies in anti-N IgG antibody response during a 10-week follow-up, with higher levels in females at weeks 6 and 7. Moreover, another study found that IgA titers were significantly higher in men than in women [69]. In Serbia, a study of COVID-19 plasma from 468 convalescent individuals found a correlation between higher levels of anti-SARS-CoV-2 antibodies and the presence of hypertension (p = 0.008) and male gender (p = 0.034) [70]. These gender disparities in immune responses are likely multifactorial, primarily influenced by sex hormones, transcription factors, and the genetic makeup of the second X chromosome [71,72]. Our study revealed that IgG/IgA antibody levels were higher in the older groups (>50) than in the younger groups. Consistent with our results, several studies have reported that advanced is associated with a high peak of convalescent antibody titers [60,70,73]. This suggests that older patients may have a stronger immune response against SARS-CoV-2. This may be attributed to increased cross-reactivity with other human coronaviruses. In this study, we examined the impact of ChAdOx1 nCoV-19 vaccination on the humoral response in patients who received the vaccine prior to infection and in those who were not vaccinated for one month after SARS-CoV-2 infection. Our findings indicate that after two weeks, antibody levels were significantly higher in the unvaccinated group than in the previously vaccinated patients (p = 0.020). Based on the results of several studies, it has been reported that COVID-19 patients and vaccinated individuals with SARS-CoV-2 messenger RNA-based or inactivated SARS-CoV-2 vaccines may have a reduced or negative IgM response [74–77]. Moreover, Xu et al recently found that pre-existing immunity can lead to suppression of the IgM response to COVID-19 vaccines [78]. In addition, Al-Tamimi et al. demonstrated that the positivity and titers of IgM response in vaccinated individuals were lower than in patients naturally infected with COVID-19 [58].

In this study, we used the Architect assay to measure anti-RBD IgG antibody levels. Previous research has indicated that assays measuring anti-RBD IgG correlate positively with SARS-CoV-2 neutralizing antibody titers [79,80]. It has also been reported that anti-RBD antibodies are responsible for most of the neutralization activity [81,82].

This study has a number of limitations. Firstly, due to limited resources, we were unable to assay the neutralizing antibody tiers and memory B and T cell responses, hence, it is difficult to correlate our results with protective antibody response or obtain information on immune memory. Secondly, several patients in our study were vaccinated, limiting the number of patients for follow-up. Despite these limitations, our data is the first in North Africa to assess the durability of humoral immunity to SARS-CoV-2 induced after natural infection.

## Conclusion

We present the first study in Africa to measure the kinetics of antibody response (IgA, IgM, and IgG) to SARS-CoV-2 over one year. Most participants remained seropositive for anti-RBD IgG after one year, but showed a significant decline in antibody titers, strongly suggesting the formation of a long-lasting immunological memory that may contribute to herd immunity. On the other hand, IgA and IgM antibody responses showed similar kinetics during the first month after SARS-CoV-2 infection, but the intensity of the IgA response was greater than that of the IgM response. To establish a link between the presence of antibodies and the level of protection against SARS-CoV-2 reinfection, the dynamic of humoral and cellular anti-SARS-CoV-2 immunity should be investigated. These results should help ministry of health to define the best vaccination strategies and the booster dose schedule.

## Supporting information

S1 Data Raw data of COVID-19 patients recruited in this study. Raw data of this study including demographic data, clinical data and Immunoglobine response (IgM, IgA, IgG). These data were used to generate Table 1 and Figs 2–10.
(XLSX)
